# Zeolite‐containing mixture alleviates microbial dysbiosis in dextran sodium sulfate‐induced colitis in mice

**DOI:** 10.1002/fsn3.2042

**Published:** 2020-11-30

**Authors:** Weida Lyu, Huijuan Jia, Chuanzong Deng, Seigo Yamada, Hisanori Kato

**Affiliations:** ^1^ Graduate School of Agricultural and Life Sciences The University of Tokyo Tokyo Japan; ^2^ Azuma Chemical Co. Ltd Tokyo Japan

**Keywords:** azumaceramics, dextran sodium sulfate, gut microbiota, inflammatory bowel disease, omics analysis, zeolite‐containing mixture (Hydryeast®)

## Abstract

Inflammatory bowel disease (IBD) is a multifactorial immunomodulatory disorder. In relative nosogenesis, gut microbiota has been the focus of research on IBD. In our previous study, we demonstrated the ameliorating effect of zeolite‐containing mixture (Hydryeast^®^, HY) on dextran sodium sulfate (DSS)‐induced colitis, through transcriptomics and proteomics. In the present study, we performed further investigation from the perspective of metagenomics using the gut microbiota. C57BL6 mice were provided an AIN‐93G basal diet or a 0.8% HY‐containing diet, and sterilized tap water for 11 days. Thereafter, colitis was induced by providing 1.5% (w/v) DSS‐containing water for 9 days. DNA was extracted from the cecal contents and pooled into libraries in a single Illumina MiSeq run. The resulting sequences were analyzed using Quantitative Insights Into Microbial Ecology (QIIME) software. According to the alterations in the relative abundance of certain bacteria, and the related gene and protein expressions, HY supplementation could improve the gut microbiota composition, ameliorate the degree of inflammation, inhibit the colonic mucosal microbial growth, and, to some extent, promote energy metabolism in the colon compared with the DSS treatment. Thus, we believe that HY may be a candidate to prevent and treat IBD.

## Graphical abstract

In our previous study, we used transcriptomics and proteomics to understand the effect of the zeolite‐containing mixture, Hydryeast® (HY) in improving dextran sodium sulfate (DSS)‐induced inflammation. In this study, we found that HY supplementation can improve the composition of gut microbiota. Moreover, we demonstrated HY may be able to improve energy metabolism in colon, resulting in DSS‐induce colitis amelioration.

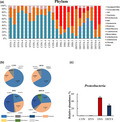

## 1 INTRODUCTION

Inflammatory bowel disease (IBD), the umbrella term used for Crohn's disease and ulcerative colitis, is induced by multiple factors including genetic predisposition, environmental factors, gut microbiota, food, and smoking (Peloquin & Nguyen, 2013; Richman & Rhodes, 2013). IBD is distinguished from other diseases by its chronic, remittent, and inflammation‐mediated disorders that affect the gastrointestinal tract (Jakubowski et al., 2014). Recently, gut microbiota research has garnered attention in human health‐related research (Peng et al., 2020; Ramli et al., 2020; Tanabe et al., 2020), and the gut microbiota is a major factor associated with IBD (Chen et al., 2017; Jakubowski et al., 2014; Oh et al., 2020). However, to the best of our knowledge, the precise etiology of IBD has not been fully elucidated, and there are only a few therapies to completely cure IBD.

Zeolite‐containing mixture, registered as Hydryeast^®^ (HY, Azuma Chemical Co., Ltd., Tokyo, Japan), is a nutraceutical, and its main ingredient is Azumaceramics (Lyu et al., 2017). Azumaceramics is a mixture of zeolite and oyster shell, burned under a high temperature. Zeolite, a microporous mineral, is utilized as an additive in the fodder of livestock and poultry, owing to its ability to improve the absorptivity and bioavailability of some minerals and vitamins (Katsoulos et al., 2009; Li, Li, et al., 2015). Zeolite has been used in industries and medical field for its cation exchange property (Tondar et al., 2014). Recently, zeolite has garnered increasing attention, especially in the field of human health, owing to its anticancer and antioxidative effects (Pavelic et al., 2001; Zarkovic et al., 2003), sustained drug‐release property (Vilaca et al., 2013), and ethanol‐absorption capacity (Federico et al., 2015).

In our previous study, we demonstrated the ameliorating effect of HY‐ on DSS‐induced colitis in mice, by integrating transcriptomics and proteomics. The results revealed that HY could improve DSS‐induced inflammation by suppressing the intestinal inflammatory pathway and ameliorating apoptosis in colon mucosa (Lyu et al., 2017). In the present study, we used nutrigenomics to gain a more comprehensive understanding of HY as a dietary supplement for improving colitis in a DSS‐induced colitis mouse model, through metagenomics of the gut microbiota.

## 2 MATERIALS AND METHODS

### 2.1 Animals and dietary treatment

Seven‐week‐old male C57BL6 mice were purchased from Charles River Japan (Tokyo, Japan) and individually maintained in cages under controlled temperature (23 ± 2°C), relative humidity (50%–60%), and lighting conditions (12‐:12‐hr light/dark cycle). After 3 days of acclimatization, the mice were divided into four groups, with equal mean body weight per group. There were 6–8 mice per group. The groups were as follows: (a) CON, provided the American Institute of Nutrition in 1993 (AIN‐93G) basal diet and sterilized tap water throughout the experiment; (b) HY8, provided the AIN‐93G basal diet supplanted with 0.8% HY powder and sterilized tap water throughout the experiment; (c) DSS, provided the AIN‐93G basal diet and sterilized tap water for 11 days, and then, colitis was induced by administering 1.5% (w/v) DSS (molecular weight, 40 kDa; MP Biomedicals, Irvine, CA, USA)‐containing sterilized tap water for 9 days; and (d) DHY8, provided the AIN‐93G basal diet supplemented with 0.8% HY powder throughout the experiment and sterilized tap water for 11 days, and then, colitis was induced by administering 1.5% DSS‐containing sterilized tap water for 9 days. The compositions of both 0.8% HY‐supplemented diet (adjusted using cornstarch to maintain the caloric balance) and the HY is are separately shown in Table S1 (A&B). We selected 0.8% as HY concentration based on our preliminary dose‐dependence experiment (unpublished data).

### 2.2 Tissue and Cecal Content Harvest

All mice were anesthetized with pentobarbital sodium before euthanasia. The colonic mucosa and cecal contents were collected and stored at −80°C until further analysis.

### 2.3 Total RNA extraction, quality assessment, and reverse transcription‐polymerase chain reaction

RNA from the total colon mucosa was extracted using the total RNA Isolation Kit, NucleoSpin^®^ RNAΠ (Macherey‐Nagel, Düren, Germany), according to the manufacturer's instructions. RNA concentration and purity were measured using a NanoDrop ND‐1000 spectrophotometer (NanoDrop Technologies, Wilmington, DE, USA). Reverse transcription‐polymerase chain reaction (RT‐PCR) was conducted to determine the relative expression of genes, and the sequence of primers used is shown in Table S2. The expression of genes in the colon mucosa was normalized against that of 60S acidic ribosomal protein p1 (*Rplp1*).

### 2.4 Cecal Bacterial DNA Extraction and Metagenomic Analysis

We performed this experiment according to a previously described protocol (Jia et al., 2017). DNA from the cecal contents for metagenomics was extracted using the QIAamp Stool Mini Kit (Qiagen, Hilden, Germany), following the manufacturer's instructions. We amplified the variable regions, 3 and 4, of the 16S rRNA gene using the primers 5′‐CCTACGGGNGGCWGCAG‐3′ and 5′‐GACTACHVGGGTATCTAATCC‐3′, followed by a modification to include the Illumina adapters and barcode sequences for sequencing. The amplification process in the DSS and DHY8 groups was not conducted smoothly; thus, the number of mice in the DSS and DHY8 groups was 5 and 7, respectively. Library size and quantification analysis were conducted using the Agilent 2,100 Bioanalyzer (Agilent Technologies, Santa Clara, CA, USA). All libraries were pooled in a single Illumina MiSeq run (MiSeq Reagent Kit V3, 600 cycles, Illumina, San Diego, CA, USA) to generate paired‐end reads of length 300 bases in each direction, following the manufacturer's instructions. Quantitative Insights Into Microbial Ecology (QIIME v1.8.3) (http:/www.qiime.org) was used to merge the overlapping paired‐end reads. The USEARCH method was used to remove the chimeric sequences against the Greengenes alignment (v gg_13_8), and reads longer than 250 bp and with an average quality score above 30 were retained.

With a threshold of 97% pairwise identity, we assigned the resulting sequences to operational taxonomic units (OTUs) and classified the representative sequences using the Ribosomal Database Project (RDP) classifier in QIIME, and then the Greengenes OUT database. We graphically visualized the distance between samples using the principal coordinate analysis (PCoA).

### 2.5 Statistical analysis

The experiment results are presented as mean ± standard error (SE). For statistical analyses of the data, p values were analyzed using the two‐way analysis of variance (ANOVA). Significant differences were evaluated using Tukey's test at *p < 0.05 and ** *p* < 0.01.

## 3 RESULTS

### 3.1 Microbiota dysbiosis and community structure

**Figure 1:**
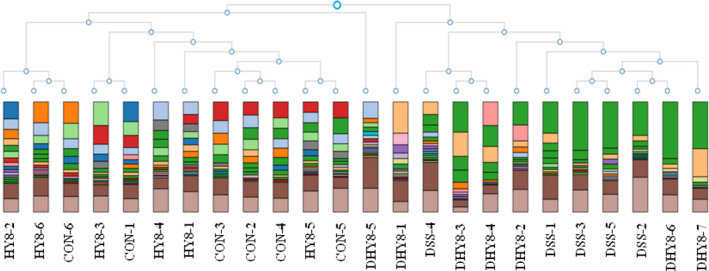
Hierarchical clustering dendrogram analysis

**Figure 2:**
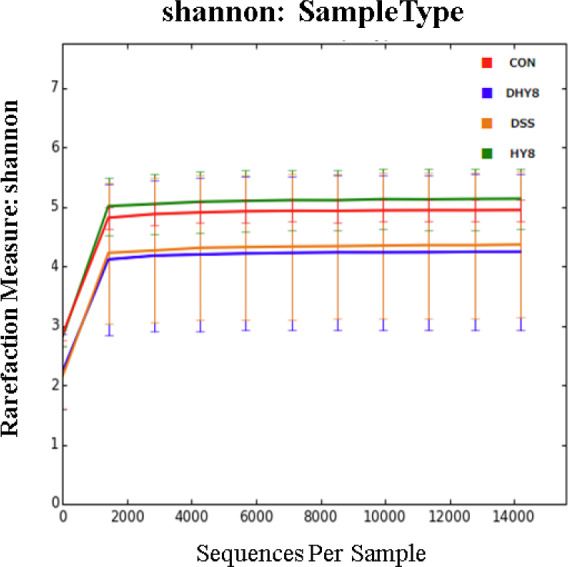
Alpha diversity analysis using the Shannon diversity index

**Figure 3:**
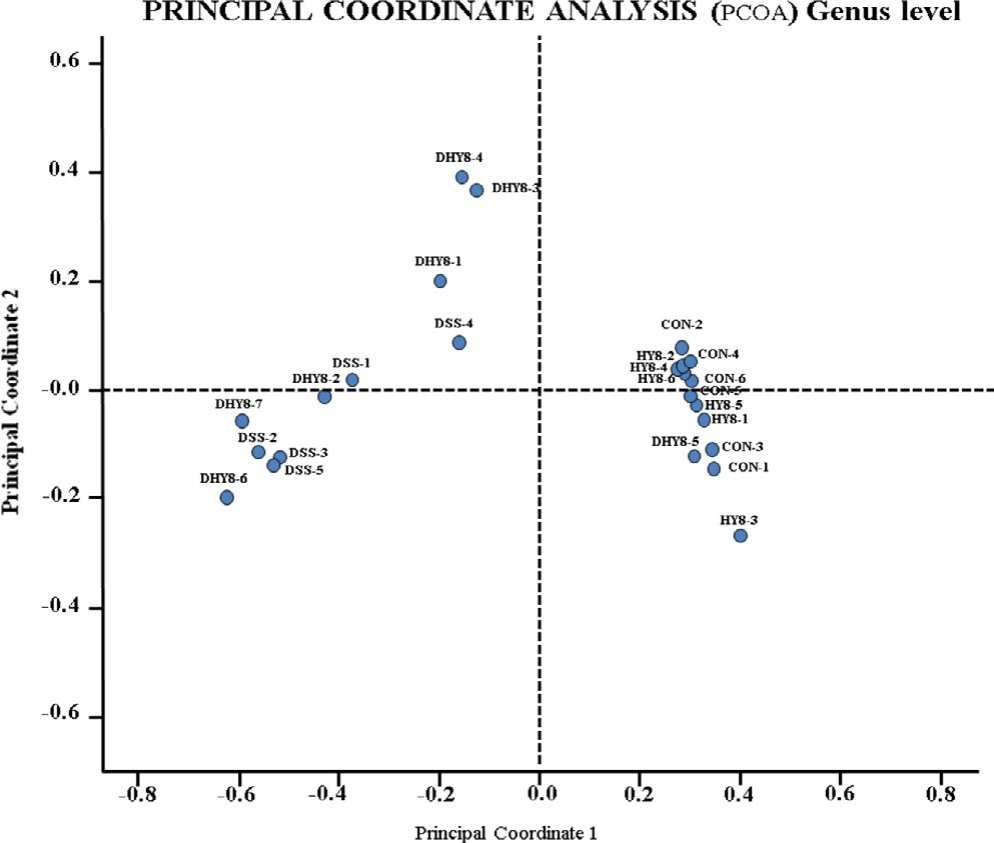
Principal coordinate analysis (PCoA) at the genus level

According to the hierarchical clustering dendrogram analysis, the gut microbiota composition of the DSS drinking groups (DSS and DHY8) was clearly different from that of the DSS nondrinking groups (CON and HY8) (Figure 1). Alpha diversity was measured using the Shannon diversity index. It showed that the DSS treatment decreased the diversity of microbiota compared with that of the CON and HY8 groups; however, no obvious difference was observed compared with that of the DHY8 group (Figure 2). The PCoA was performed to measure the differences in the distribution of taxonomic classifications between samples, up to a fixed taxonomic level. The results of the PCoA at the genus level showed that microbial distribution in some mice in the DHY8 group was closer to that in the CON and HY8 groups than the DSS group (Figure 3).

**Figure 4:**
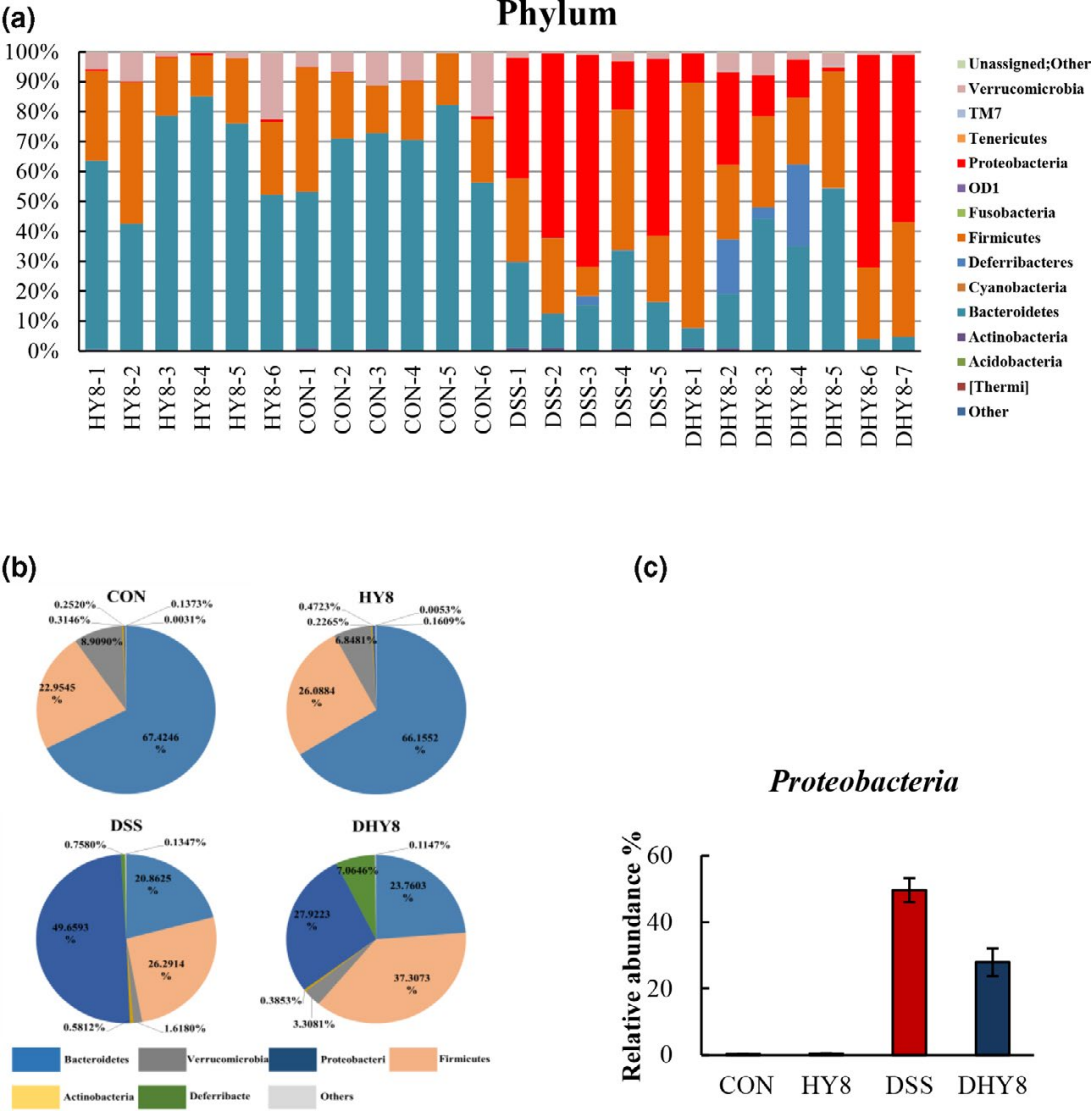
A, Comparative analysis of the taxonomic composition of the microbial community at the phylum level. B, Phylum level of microbiota profiling. C, The relative abundance of the proinflammation‐related bacteria, Proteobacteria members, at the phylum level. All values are presented as mean ± SE (*n* = 5–7) by the two‐way ANOVA and Tukey's test; *p < 0.05 and **p < 0.01

**Figure 5:**
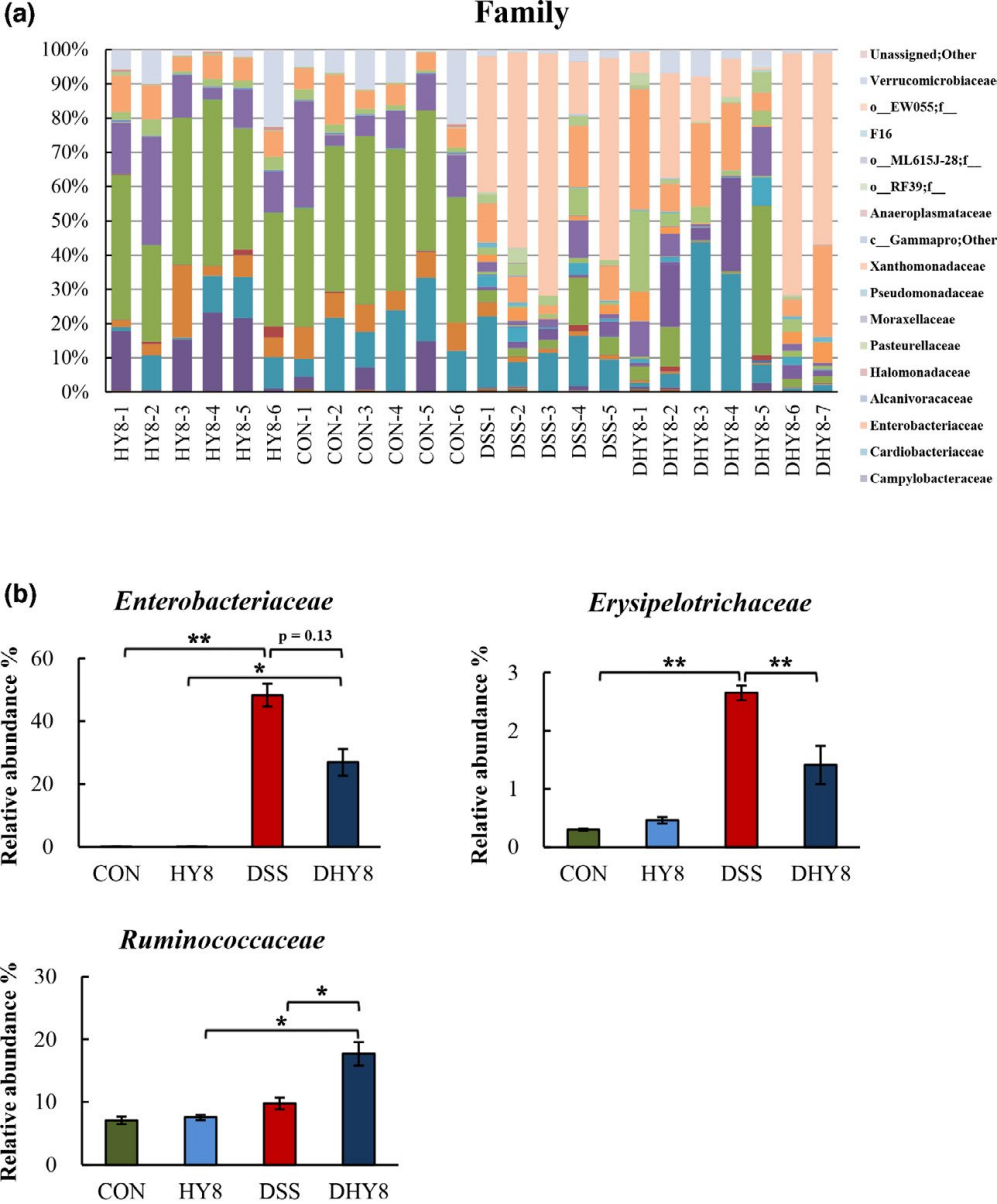
A, Comparative analysis of the taxonomic composition of the microbial community at the family level. (f_ means unassigned bacteria). B, The relative abundance of IBD marker and the inflammation‐related bacteria, Enterobacteriaceae and Erysipelotrichaceae members, and the SCFA‐related bacteria, Ruminococcaceae members, at the family level. All values are presented as mean ± SE (*n* = 5–7) by the two‐way ANOVA and Tukey's test; *p < 0.05 and **p < 0.01

**Figure 6:**
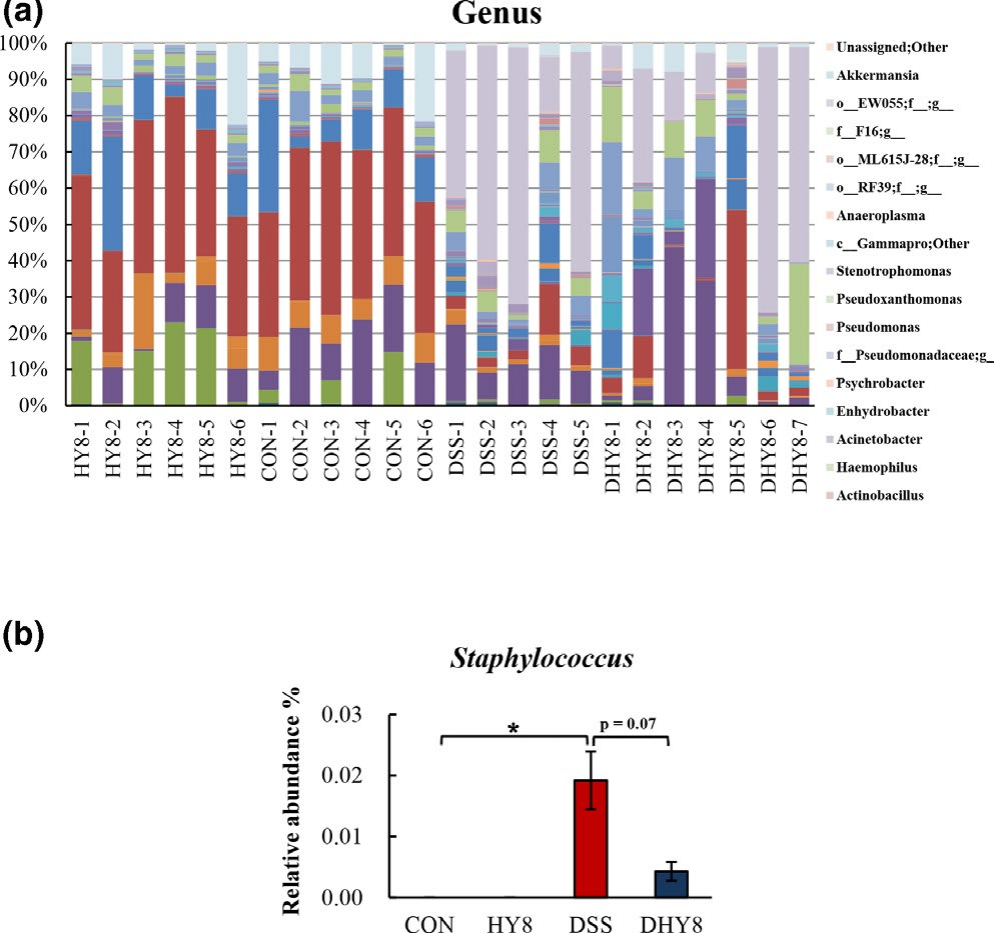
A, Comparative analysis of the taxonomic composition of the microbial community at the genus level. (f_ and g_ mean unassigned bacteria). B, The relative abundance of a marker bacterium of IBD, *Staphylococcus*, at the genus level. All values are presented as mean ± SE (*n* = 5–7) by two‐way ANOVA and Tukey's test; *p < 0.05 and **p < 0.01

**Figure 7:**
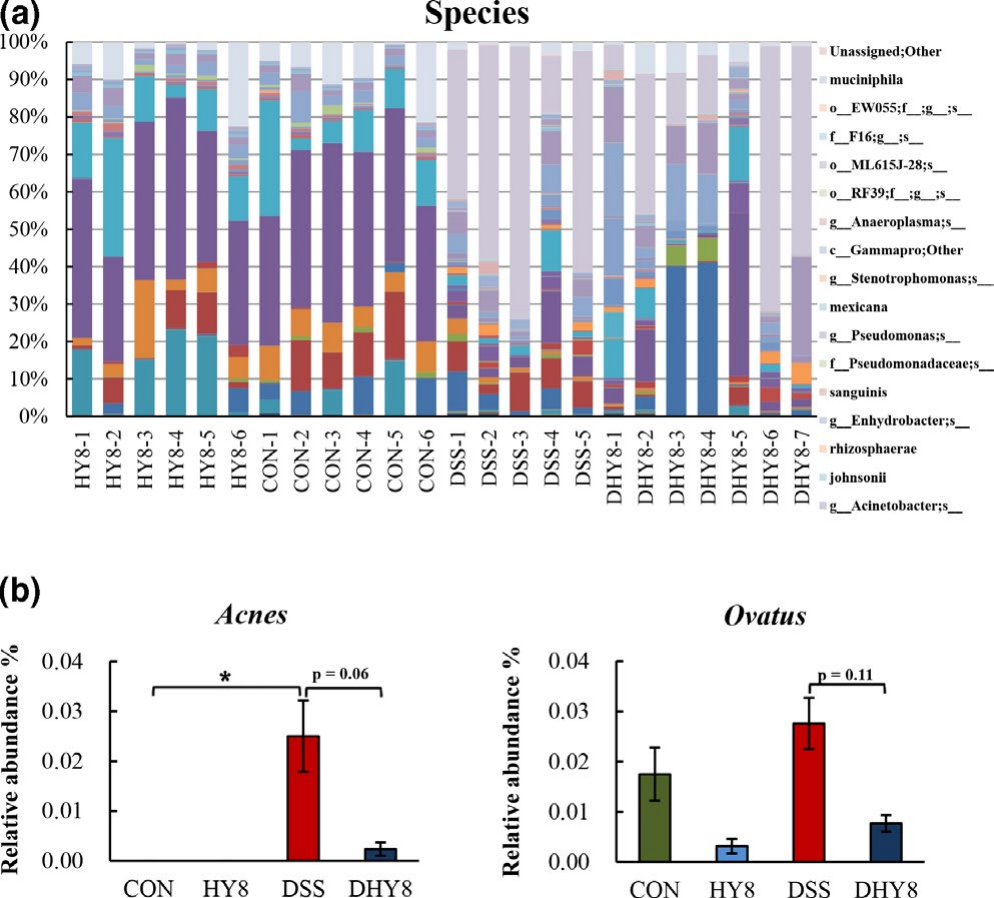
A, Comparative analyses of the taxonomic composition of the microbial community at the species level. (f_, g_, and s_ mean unassigned bacteria). B, The relative abundance of the bacteria at the species level: immune system and inflammation‐related bacteria, *P. acnes*, and the antibody response‐related bacteria, *Bacteroides ovatus*. All values are presented as mean ± SE (*n* = 5–7) by the two‐way ANOVA and Tukey's test; *p < 0.05 and **p < 0.01

A comparative analysis of the taxonomic composition of the microbial community at the phylum level was achieved as shown in Figure 4a. The phylum level of microbiota profiling is shown in Figure 4b. The relative abundance of the proinflammation‐related bacterial phylum, Proteobacteria, ranged from 0.25% in the CON group and 0.47% in the HY8 group to 49.66% in the DSS group and 27.92% in the DHY8 group (Figure 4c). The results of the comparative analyses of the taxonomic composition of the microbial community at the family level are shown in Figure 5a. HY supplementation altered the relative abundance of some bacteria. The marker bacteria of IBD, Enterobacteriaceae members, ranged from 0.06% in the CON group and 0.04% in the HY8 group to 48.28% in the DSS group and 26.87% in the DHY8 group. The inflammation‐related bacteria, Erysipelotrichaceae members, ranged from 0.46% in the CON group and 0.30% in the HY8 group to 2.65% in the DSS group and 1.41% in the DHY8 group. HY supplementation significantly downregulated the relative abundance of bacteria in the DHY8 group compared with that in the DSS group. The short chain fatty acid (SCFA)‐related bacteria, Ruminococcaceae members, ranged from 7.55% and 7.10% in the CON and HY8 groups, respectively, to 9.78% and 17.70% in the DSS and DHY8 groups, respectively (Figure 5b). The relative abundance of these bacteria in the DHY8 group was significantly increased compared with that in the DSS group. At the genus level (Figure 6a), the relative abundance of a marker bacterium of IBD, *Staphylococcus*, ranged from 0.000% and 0.000% in the CON and HY8 groups, respectively, to 0.019% and 0.004% in the DSS and DHY8 groups, respectively (Figure 6b). At the species level (Figure 7a), the relative abundance of the inflammation‐related bacterium, *Propionibacterium acnes*, ranged from 0.000% and 0.000% in the CON and HY8 groups, respectively, to 0.025% and 0.002% in the DSS and DHY8 groups, respectively (Figure 7b).

### 3.2 Gene expression in the colonic mucosa

**Figure 8:**
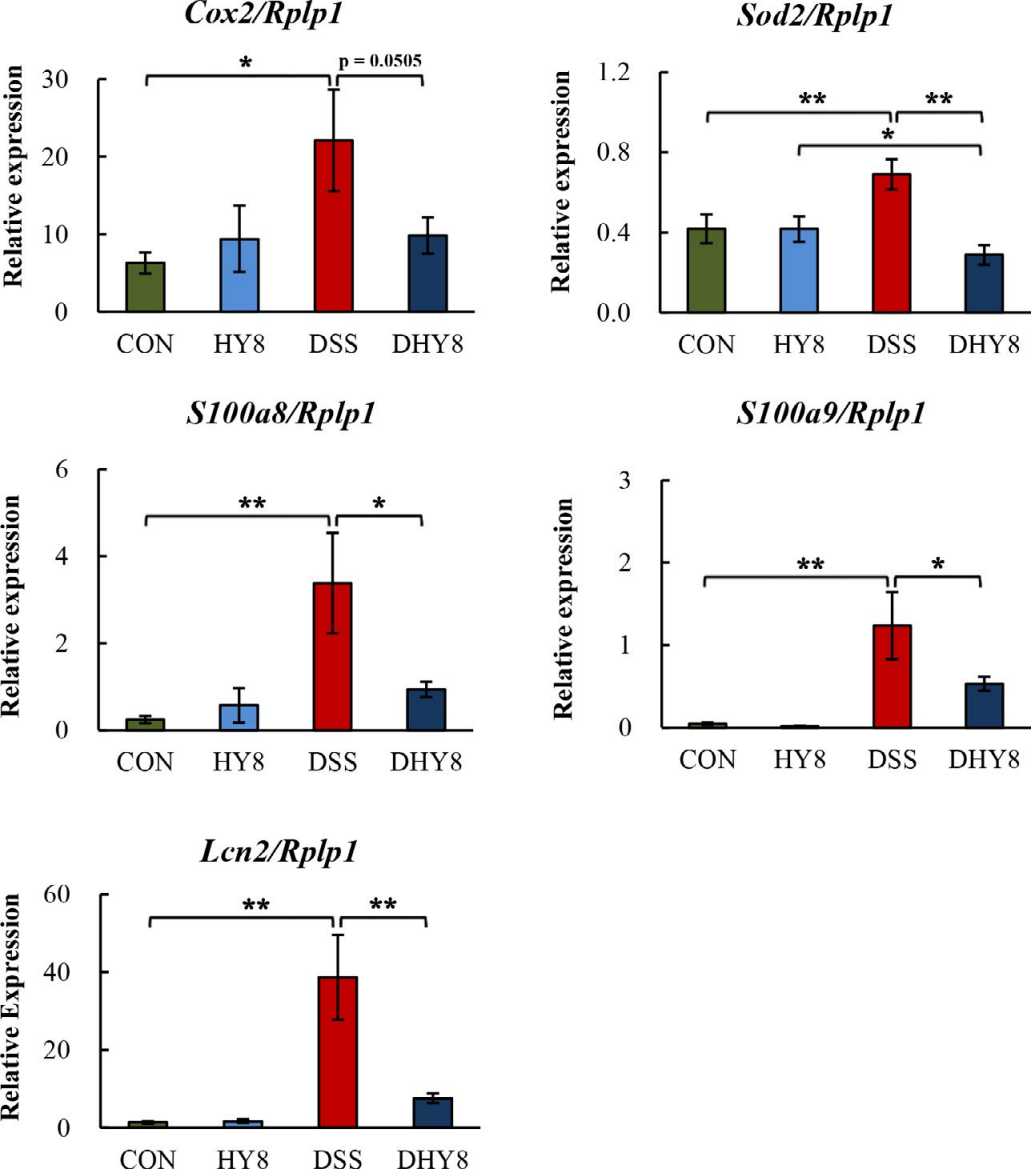
mRNA expression of colonic mucosal genes. The relative mRNA expression of *Cox2*, *Sod2*, *Lcn2*, *S100a8*, and *S100a9* was measured by RT‐PCR and normalized to that of *Rplp1*. All values are presented as mean ± SE (*n* = 5–7) by the two‐way ANOVA and Tukey's test; *p < 0.05 and **p < 0.01

HY supplementation downregulated the expression of inflammation regulator and mediator gene, superoxide dismutase 2 (*Sod2*), oxidative stress‐related gene, cytochrome c oxidase subunit 2 (*Cox2*), microbial growth‐related genes, lipocalin 2 (*Lcn2*), s100 calcium‐binding protein A8 (*S100a8*), and s100 calcium‐binding protein A9 (*S100a9*) in the DHY8 group compared with the DSS treatment (Figure 8).

## 4 DISCUSSION

The alpha diversity results revealed no significant effect of HY supplementation on microbiota diversity. However, the PCoA analysis suggested that HY supplementation improved the gut microbiota composition.

The supplementation of HY significantly ameliorated the inflammation condition by altering the relative abundance of the gut bacteria. Proteobacteria, at the phylum level, showed the tendency to decrease with HY supplementation. Thus, members of this phylum may have unique abilities that allow them to exploit host defenses and promote proinflammatory changes in susceptible hosts (Mukhopadhya et al., 2012). Here, we found that the relative abundance of some IBD‐related bacteria was altered by HY supplementation. The relative abundance of the marker bacteria of IBD, Erysipelotrichaceae members, at the family level was significantly decreased in the HY8 group compared with that in the DSS group. It has been reported that inflammation associated with colitis increases the abundance of Erysipelotrichaceae members; thus, limiting the expansion of Erysipelotrichaceae members will improve colitis (Chen et al., 2017). Erysipelotrichaceae members have also been associated with an elevation in TNF and chronic intestinal inflammation in animals infected with simian immunodeficiency virus (Handley et al., 2016). In addition, Enterobacteriaceae comprises several pathogenic bacterial families and is a marker of intestinal inflammation and oxidative stress in human IBD and murine colitis (Jia et al., 2017). It has been reported that zeolite supplementation in diet could reduce the abundance of the pathogen‐rich bacteria, Enterobacteria, in poultry (Prasai et al., 2017). In the present study, we found a decreasing tendency in the relative abundance of Enterobacteriaceae members in the DHY8 group compared with that in the DSS group (*p = 0.13*).

At the genus level, the decrease in the relative abundance of *Staphylococcus* spp. indicated that HY supplementation improved DSS‐induced colitis. *Staphylococcus* is implicated as a major cause of antibiotic‐associated enterocolitis and antibiotic‐associated diarrhea (Altemeier et al., 1963; Khan & Hall, 1966) and is also defined as the diagnosis *Staphylococcus aureus* (*S. aureus*) enterocolitis (Boyce & Havill, 2005). Additionally, supplementing HY decreased the relative abundance of *P. acnes* at the species level. *Propionibacterium acnes* is a normal inhabitant of the gastrointestinal tract and is also related to the immune system; it may cause a granulomatous reaction (Wada et al., 2001). *Propionibacterium acnes* has been identified as the causative agent of granulomatous lesions in soft tissue inflammation, meningitis, pneumonia, and hepatitis (Esteban et al., 1994). The overgrowth of *P. acnes* in Crohn's disease has also been reported (Bashir et al., 2016). *Bacteroides ovatus* has been proven to injure the gut tissue, resulting in the induction of inflammation, and an accompanying elevation in serum antibodies in patients with IBD (Saitoh et al., 2002). However, in the present study, the relative abundance of *B. ovatus* decreased with HY supplementation in the HY8 and DHY8 groups compared with that in the DSS group.

Besides the alterations in the bacteria mentioned above, the alleviated inflammatory status can also be attributed to the relative expression of genes in the colon mucosa. HY supplementation downregulated the expression of *Cox2*, which is an inflammatory regulator and mediator (Zhang et al., 2014). The infiltration of neutrophils and macrophages in the colon suggested an inflammatory response. In addition, inflammatory cytokines were produced by the activated macrophages (Xavier & Podolsky, 2007). In oral keratinocytes, Staphylococcus spp., especially *S. aureus*, could induce infection‐associated malignant transformation of oral epitheliums via *Cox2* activation (Wang et al., 2019). *Staphylococcus* aureus can also upregulate *Cox2* expression and prostaglandin E2 (*Pge2*) secretion in human aortic endothelial cells and induce *Pge2*/interleukin 6 (*Il‐6*)/matrix metallopeptidase‐9‐dependent cell migration (Tsai et al., 2018). Moreover, it has been reported that the expression of *Cox2* and the subsequent production of nitric oxide and prostaglandin increased after *P. acnes* infection in HaCaT cells; *P. acnes* also induced the expression of interleukin 1 beta (*Il‐1β*) (Jin & Lee, 2018). According to the above indirect evidence, we speculated that HY supplementation could decrease the relative abundance of Staphylococcus and then downregulate the inflammation‐related genes *Cox2* and *Il‐6*. Moreover, a reduction in the relative abundance of *P. acnes* suppressed the expression of *Cox2* and *Il‐1β*. The expression of the inflammation‐related genes *Il‐6*, *Il‐1β*, and tumor necrosis factor‐alpha (*Tnf‐α*) was investigated in our previous study (Lyu et al., 2017).

*Sod2* encodes a mitochondrial enzyme that quenches free radicals and protects against oxidative stress by converting superoxide radicals to H_2_O_2_ (Kosaka et al., 2009). In the present study, under oxidative stress, the expression of *Sod2* was downregulated by HY supplementation. It has been reported that *S. aureus* peritonitis can significantly induce *Sod2* in WT mice (Cherry et al., 2014). The host response to *S. aureus* involves the generation of sufficient amounts of *Tnf‐α* and *Il‐1β* (Hattar et al., 2006). Similar alterations were observed in our study, indicating the HY supplementation might alleviate oxidative stress and inflammation by suppressing the relative abundance of *S. aureus* and then the expression of *Sod2*, *Tnf‐α*, and *Il‐1β*. In our previous study, we proposed that supplementing HY may ameliorate DSS‐induced inflammation by downregulating the expressions of genes in the intestinal inflammatory pathway. The expression of the proinflammatory cytokine genes, *Il‐6*, *Il‐1β*, and *Tnf‐α,* was suppressed. Moreover, upregulated expression of anti‐inflammation‐related proteins including adenylate cyclase‐activating polypeptide and galectin 2 may contribute to this amelioration. Similarly, downregulating inflammation degree‐related proteins such as haptoglobin, complement component 3, myeloperoxidase, Annexin A2, heat shock protein family A member 4, and heterogeneous nuclear ribonucleoprotein U (Hnrpu), a protein biomarker for colorectal carcinoma, may also assist in improving DSS‐induced inflammation (Lyu et al., 2017).

The downregulation of the following genes also indicated that HY supplementation inhibited bacterial growth in the colon. (a) *S100a8* and *S100a9*, two monomers of calprotectin that could sequester manganese and zinc ions, and thus limit the growth of bacteria (Faber & Baumler, 2014). The extracellular S100a8 and S100a9 proteins increase the levels of diverse inflammatory mediators via TLR‐4 engagement, and these proteins are released from neutrophils and the extracellular matrix in the presence of *S. aureus* (Van Crombruggen et al., 2016). A similar association between *S100a8/S100a9* and *S. aureus* was also observed in our study. (b) *Lcn2*, which could prevent bacterial iron acquisition by binding and sequestering enterobactin (Flo et al., 2004), and may affect the growth of bacteria. Thus, the altered expression of these genes indicates that HY supplementation could inhibit bacterial growth induced by DSS. The abundance of Proteobacteria members in fecal bacterial composition reportedly decreased in *Lcn2* knockout (KO) mice compared with that in WT mice (Singh et al., 2016). On the contrary, Proteobacteria members, regarded as opportunistic, colitogenic, and a marker of dysbiosis, moderately increased in *Lcn2* KO mice; it is necessary to further investigate the effect of *Lcn2* on gut microbiota and mucosal barrier function in the colon (Toyonaga et al., 2016). *Lcn2* is also known to be overexpressed in human colorectal cancer (CRC) and other cancers (Maier et al., 2014). It is negatively correlated with the decrease in Ruminococcaceae members in CRC (Burns et al., 2015; Peters et al., 2016). In our study, HY supplementation significantly suppressed the expression of *Lcn2* and improved the relative abundance of Ruminococcaceae members; these alterations might help improve energy metabolism as mentioned above.

In this study, Ruminococcaceae members attracted our attention owing to their association with the production of SCFA. Ruminococcaceae members may release ammonia or amine, and via the enteric fermentation of SCFAs, promote the ratio of carbohydrate‐to‐nitrogen as fermentative substrates. This in turn may increase SCFA availability, leading to an improvement in energy metabolism (Jia et al., 2017). In the present study, the relative abundance of Ruminococcaceae members was significantly increased by HY supplementation in the DHY8 group compared with that in the DSS group. However, their relative abundance in the DSS group was not significantly decreased compared with that in the CON group. The alteration in the abundance of Ruminococcaceae members suggests, to some extent, that HY supplementation improved energy metabolism. The proteome analysis revealed upregulation of the energy utilization‐related protein fatty acid‐binding protein (Fabp4) in adipocytes (fold change for DSS versus. CON: 0.06 and DHY8 versus. DSS: 7.73). Fabp4, a member intracellular fatty acid‐binding protein family, plays a vital role in the uptake of fatty acids and in intracellular transport (Bernlohr et al., 1985; Li, Zhao, et al., 2015). Fabp4 is also a key contributor in maintaining systemic glucose metabolism and the biology of adipocytes (Scheja et al., 1999; Uysal et al., 2000). The above results suggest that HY supplementation may have the potential to improve energy metabolism and thus ameliorate DSS‐induced colitis.

## 5 CONCLUSIONS

Our observations of the gut microbiota and the relative alterations in gene expression in the colon mucosa revealed that HY supplementation could ameliorate DSS‐induced colitis in mice by improving the inflammation status, limiting the growth of bacteria in gut, and improving energy metabolism. Furthermore, we conducted the correlation analysis between microbial markers and colonic mucosal genes. Concomitantly, in our previous study, we demonstrated that HY supplementation could ameliorate DSS‐induced inflammation and apoptosis in the colon, using transcriptomics and proteomics. Overall, we believe that Hydryeast is a promising candidate to prevent and treat IBD.

## CONFLICT OF INTEREST

CD and SY are employees of Azuma Chemical Co., Ltd. All the other authors declare no competing interests.

## AUTHOR CONTRIBUTIONS

The study was designed by H.J. The experiments and data collection were performed by H.J., C.D., and W.L. Data were analyzed by H.J., C.D., and W.L. The paper was written by W.L. and revised by H.K. and H.J. The experimental materials were provided by S.Y. All authors have reviewed the manuscript.

## ETHICAL APPROVAL

Ethical review: This research was approved by the Animal Care and Use Committee of the University of Tokyo (Approval No. P13‐739).

## Supporting information

TableS1‐S2

## References

[fsn32042-bib-0001] Altemeier, W. A. , Hummel, R. P. , & Hill, E. O. (1963). Staphylococcal enterocolitis following antibiotic therapy. Annals of Surgery, 157, 847–858. Retrieved from https://www.ncbi.nlm.nih.gov/pubmed/14012299. 10.1097/00000658-196306000-00003 14012299 PMC1466478

[fsn32042-bib-0002] Bashir, A. , Miskeen, A. Y. , Hazari, Y. M. , Asrafuzzaman, S. , & Fazili, K. M. (2016). *Fusobacterium nucleatum*, inflammation, and immunity: The fire within human gut. Tumour Biology, 37(3), 2805–2810. 10.1007/s13277-015-4724-0 26718210

[fsn32042-bib-0003] Bernlohr, D. A. , Doering, T. L. , Kelly, T. J. Jr , & Lane, M. D. (1985). Tissue specific expression of p422 protein, a putative lipid carrier, in mouse adipocytes. Biochemical and Biophysical Research Communications, 132(2), 850–855. Retrieved from https://www.ncbi.nlm.nih.gov/pubmed/2415129. 10.1016/0006-291X(85)91209-4 2415129

[fsn32042-bib-0004] Boyce, J. M. , & Havill, N. L. (2005). Nosocomial antibiotic‐associated diarrhea associated with enterotoxin‐producing strains of methicillin‐resistant *Staphylococcus aureus* . American Journal of Gastroenterology, 100(8), 1828–1834. 10.1111/j.1572-0241.2005.41510.x 16086721

[fsn32042-bib-0005] Burns, M. B. , Lynch, J. , Starr, T. K. , Knights, D. , & Blekhman, R. (2015). Virulence genes are a signature of the microbiome in the colorectal tumor microenvironment. Genome Medicine, 7(1), 55 10.1186/s13073-015-0177-8 26170900 PMC4499914

[fsn32042-bib-0006] Chen, L. , Wilson, J. E. , Koenigsknecht, M. J. , Chou, W.‐C. , Montgomery, S. A. , Truax, A. D. , Brickey, W. J. , Packey, C. D. , Maharshak, N. , Matsushima, G. K. , Plevy, S. E. , Young, V. B. , Sartor, R. B. , & Ting, J.‐Y. (2017). NLRP12 attenuates colon inflammation by maintaining colonic microbial diversity and promoting protective commensal bacterial growth. Nature Immunology, 18(5), 541–551. 10.1038/ni.3690 28288099 PMC5395345

[fsn32042-bib-0007] Cherry, A. D. , Suliman, H. B. , Bartz, R. R. , & Piantadosi, C. A. (2014). Peroxisome proliferator‐activated receptor gamma co‐activator 1‐alpha as a critical co‐activator of the murine hepatic oxidative stress response and mitochondrial biogenesis in *Staphylococcus aureus* sepsis. Journal of Biological Chemistry, 289(1), 41–52. 10.1074/jbc.M113.512483 PMC3879563 24253037

[fsn32042-bib-0008] Esteban, J. , Cuenca‐Estrella, M. , Ramos, J. M. , & Soriano, F. (1994). Granulomatous infection due to *Propionibacterium* acnes mimicking malignant disease. European Journal of Clinical Microbiology and Infectious Diseases, 13(12), 1084 Retrieved from https://www.ncbi.nlm.nih.gov/pubmed/7889976. 10.1007/BF02111835 7889976

[fsn32042-bib-0009] Faber, F. , & Baumler, A. J. (2014). The impact of intestinal inflammation on the nutritional environment of the gut microbiota. Immunol Lett, 162(2 Pt A), 48–53. 10.1016/j.imlet.2014.04.014. 24803011 PMC4219934

[fsn32042-bib-0010] Federico, A. , Dallio, M. , Gravina, A. G. , Iannotta, C. , Romano, M. , Rossetti, G. , & Loguercio, C. (2015). A pilot study on the ability of clinoptilolite to absorb ethanol in vivo in healthy drinkers: Effect of gender. Journal of Physiology and Pharmacology, 66(3), 441–447. Retrieved from https://www.ncbi.nlm.nih.gov/pubmed/26084226. 26084226

[fsn32042-bib-0011] Flo, T. H. , Smith, K. D. , Sato, S. , Rodriguez, D. J. , Holmes, M. A. , Strong, R. K. , Akira, S. , & Aderem, A. (2004). Lipocalin 2 mediates an innate immune response to bacterial infection by sequestrating iron. Nature, 432(7019), 917–921. 10.1038/nature03104 15531878

[fsn32042-bib-0012] Handley, S. A. , Desai, C. , Zhao, G. , Droit, L. , Monaco, C. L. , Schroeder, A. C. , Nkolola, J. P. , Norman, M. E. , Miller, A. D. , Wang, D. , Barouch, D. H. , & Virgin, H. W. (2016). SIV infection‐mediated changes in gastrointestinal bacterial microbiome and virome are associated with immunodeficiency and prevented by vaccination. Cell Host & Microbe, 19(3), 323–335. 10.1016/j.chom.2016.02.010 26962943 PMC4802495

[fsn32042-bib-0013] Hattar, K. , Grandel, U. , Moeller, A. , Fink, L. , Iglhaut, J. , Hartung, T. , & Sibelius, U. (2006). Lipoteichoic acid (LTA) from Staphylococcus aureus stimulates human neutrophil cytokine release by a CD14‐dependent, Toll‐like‐receptor‐independent mechanism: Autocrine role of tumor necrosis factor‐[alpha] in mediating LTA‐induced interleukin‐8 generation. Critical Care Medicine, 34(3), 835–841. 10.1097/01.ccm.0000202204.01230.44 16521278

[fsn32042-bib-0014] Jakubowski, A. , Zagorowicz, E. , Kraszewska, E. , & Bartnik, W. (2014). Rising hospitalization rates for inflammatory bowel disease in Poland. Polskie Archiwum Medycyny Wewnetrznej, 124(4), 180–190. Retrieved from https://www.ncbi.nlm.nih.gov/pubmed/24727650. 10.20452/pamw.2188 24727650

[fsn32042-bib-0015] Jia, H. , Hanate, M. , Aw, W. , Itoh, H. , Saito, K. , Kobayashi, S. , Hachimura, S. , Fukuda, S. , Tomita, M. , Hasebe, Y. , & Kato, H. (2017). Eggshell membrane powder ameliorates intestinal inflammation by facilitating the restitution of epithelial injury and alleviating microbial dysbiosis. Scientific Reports, 7, 43993 10.1038/srep43993 28272447 PMC5341015

[fsn32042-bib-0016] Jin, S. , & Lee, M. Y. (2018). The ameliorative effect of hemp seed hexane extracts on the *Propionibacterium acnes*‐induced inflammation and lipogenesis in sebocytes. PLoS One, 13(8), e0202933 10.1371/journal.pone.0202933 30148860 PMC6110517

[fsn32042-bib-0017] Katsoulos, P. D. , Zarogiannis, S. , Roubies, N. , & Christodoulopoulos, G. (2009). Effect of long‐term dietary supplementation with clinoptilolite on performance and selected serum biochemical values in dairy goats. American Journal of Veterinary Research, 70(3), 346–352. 10.2460/ajvr.70.3.346 19254146

[fsn32042-bib-0018] Khan, M. Y. , & Hall, W. H. (1966). Staphylococcal enterocolitis–treatment with oral vancomycin. Annals of Internal Medicine, 65(1), 1–8. Retrieved from https://www.ncbi.nlm.nih.gov/pubmed/5936663. 10.7326/0003-4819-65-1-1 5936663

[fsn32042-bib-0019] Kosaka, T. , Yoshino, J. , Inui, K. , Wakabayashi, T. , Kobayashi, T. , Watanabe, S. , Hayashi, S. , Hirokawa, Y. , Shiraishi, T. , Yamamoto, T. , Tsuji, M. , Katoh, T. , & Watanabe, M. (2009). Involvement of NAD(P)H:Quinone oxidoreductase 1 and superoxide dismutase polymorphisms in ulcerative colitis. DNA and Cell Biology, 28(12), 625–631. 10.1089/dna.2009.0877 19715479

[fsn32042-bib-0020] Li, A. , Zhao, Z. , Zhang, Y. , Fu, C. , Wang, M. , & Zan, L. (2015). Tissue expression analysis, cloning, and characterization of the 5'‐regulatory region of the bovine fatty acid binding protein 4 gene. Journal of Animal Science, 93(11), 5144–5152. 10.2527/jas.2015-9378 26641034

[fsn32042-bib-0021] Li, L. , Li, P. , Chen, Y. , Wen, C. , Zhuang, S. , & Zhou, Y. (2015). Zinc‐bearing zeolite clinoptilolite improves tissue zinc accumulation in laying hens by enhancing zinc transporter gene mRNA abundance. Animal Science Journal, 86(8), 782–789. 10.1111/asj.12358 25597922

[fsn32042-bib-0022] Lyu, W. , Jia, H. , Deng, C. , Saito, K. , Yamada, S. , & Kato, H. (2017). Zeolite‐containing mixture supplementation ameliorated dextran sodium sulfate‐induced colitis in mice by suppressing the inflammatory bowel disease pathway and improving apoptosis in colon mucosa. Nutrients, 9(5), 10.3390/nu9050467 PMC5452197 28481231

[fsn32042-bib-0023] Maier, H. T. , Aigner, F. , Trenkwalder, B. , Zitt, M. , Vallant, N. , Perathoner, A. , Margreiter, C. , Moser, P. , Pratschke, J. , & Amberger, A. (2014). Up‐regulation of neutrophil gelatinase‐associated lipocalin in colorectal cancer predicts poor patient survival. World Journal of Surgery, 38(8), 2160–2167. 10.1007/s00268-014-2499-x 24682311

[fsn32042-bib-0024] Mukhopadhya, I. , Hansen, R. , El‐Omar, E. M. , & Hold, G. L. (2012). IBD‐what role do Proteobacteria play? Nature Reviews Gastroenterology & Hepatology, 9(4), 219–230. 10.1038/nrgastro.2012.14 22349170

[fsn32042-bib-0025] Oh, W. S. , Jung, J. C. , Choi, Y. M. , Mun, J. Y. , Ku, S. K. , & Song, C. H. (2020). Protective effects of fermented rice extract on ulcerative colitis induced by dextran sodium sulfate in mice. Food Sciences and Nutrition, 8(3), 1718–1728. 10.1002/fsn3.1460 PMC7063356 32180979

[fsn32042-bib-0026] Pavelić, K. , Hadžija, M. , Bedrica, L. , Pavelić, J. , Ðikić, I. , Katić, M. , Kralj, M. , Bosnar, M. H. , Kapitanović, S. , Poljak‐Blaži, M. , Križanac, Š. , Stojković, R. , Jurin, M. , Subotić, B. , & Čolić, M. (2001). Natural zeolite clinoptilolite: New adjuvant in anticancer therapy. Journal of Molecular Medicine (Berlin), 78(12), 708–720. Retrieved from https://www.ncbi.nlm.nih.gov/pubmed/11434724. 10.1007/s001090000176 11434724

[fsn32042-bib-0027] Peloquin, J. M. , & Nguyen, D. D. (2013). The microbiota and inflammatory bowel disease: Insights from animal models. Anaerobe, 24, 102–106. 10.1016/j.anaerobe.2013.04.006 23603043 PMC3766478

[fsn32042-bib-0028] Peng, L. , Zhang, Q. U. , Zhang, Y. , Yao, Z. , Song, P. , Wei, L. , Zhao, G. , & Yan, Z. (2020). Effect of tartary buckwheat, rutin, and quercetin on lipid metabolism in rats during high dietary fat intake. Food Sciences and Nutrition, 8(1), 199–213. 10.1002/fsn3.1291 PMC6977491 31993146

[fsn32042-bib-0029] Peters, B. A. , Dominianni, C. , Shapiro, J. A. , Church, T. R. , Wu, J. , Miller, G. , Yuen, E. , Freiman, H. , Lustbader, I. , Salik, J. , Friedlander, C. , Hayes, R. B. , & Ahn, J. (2016). The gut microbiota in conventional and serrated precursors of colorectal cancer. Microbiome, 4(1), 69 10.1186/s40168-016-0218-6 28038683 PMC5203720

[fsn32042-bib-0030] Prasai, T. P. , Walsh, K. B. , Bhattarai, S. P. , Midmore, D. J. , Van, T. T. , Moore, R. J. , & Stanley, D. (2017). Zeolite food supplementation reduces abundance of enterobacteria. Microbiological Research, 195, 24–30. 10.1016/j.micres.2016.11.006 28024523

[fsn32042-bib-0031] Ramli, N. S. , Jia, H. , Sekine, A. , Lyu, W. , Furukawa, K. , Saito, K. , Hasebe, Y. , & Kato, H. (2020). Eggshell membrane powder lowers plasma triglyceride and liver total cholesterol by modulating gut microbiota and accelerating lipid metabolism in high‐fat diet‐fed mice. Food Sciences and Nutrition, 8(5), 2512–2523. 10.1002/fsn3.1545 PMC7215208 32405407

[fsn32042-bib-0032] Richman, E. , & Rhodes, J. M. (2013). Review article: Evidence‐based dietary advice for patients with inflammatory bowel disease. Alimentary Pharmacology & Therapeutics, 38(10), 1156–1171. 10.1111/apt.12500 24102340

[fsn32042-bib-0033] Saitoh, S. , Noda, S. , Aiba, Y. , Takagi, A. , Sakamoto, M. , Benno, Y. , & Koga, Y. (2002). Bacteroides ovatus as the predominant commensal intestinal microbe causing a systemic antibody response in inflammatory bowel disease. Clinical and Diagnostic Laboratory Immunology, 9(1), 54–59. Retrieved from https://www.ncbi.nlm.nih.gov/pubmed/11777829. 10.1128/CDLI.9.1.54-59.2002 11777829 PMC119885

[fsn32042-bib-0034] Scheja, L. , Makowski, L. , Uysal, K. T. , Wiesbrock, S. M. , Shimshek, D. R. , Meyers, D. S. , Morgan, M. , Parker, R. A. , & Hotamisligil, G. S. (1999). Altered insulin secretion associated with reduced lipolytic efficiency in aP2‐/‐ mice. Diabetes, 48(10), 1987–1994. Retrieved from https://www.ncbi.nlm.nih.gov/pubmed/10512363. 10.2337/diabetes.48.10.1987 10512363

[fsn32042-bib-0035] Singh, V. , Yeoh, B. S. , Chassaing, B. , Zhang, B. , Saha, P. , Xiao, X. , Awasthi, D. , Shashidharamurthy, R. , Dikshit, M. , Gewirtz, A. , & Vijay‐Kumar, M. (2016). Microbiota‐inducible innate immune, siderophore binding protein lipocalin 2 is critical for intestinal homeostasis. Cellular and Molecular Gastroenterology and Hepatology, 2(4), 482–498 e486. 10.1016/j.jcmgh.2016.03.007 27458605 PMC4957954

[fsn32042-bib-0036] Tanabe, K. , Okuda, A. , Ken, F. , Yamanaka, N. , Nakamura, S. , & Oku, T. (2020). Metabolic fate of newly developed nondigestible oligosaccharide, maltobionic acid, in rats and humans. Food Sciences and Nutrition, 8(7), 3610–3616. 10.1002/fsn3.1643 PMC7382184 32724623

[fsn32042-bib-0037] Tondar, M. , Parsa, M. J. , Yousefpour, Y. , Sharifi, A. M. , & Shetab‐Boushehri, S. V. (2014). Feasibility of clinoptilolite application as a microporous carrier for pH‐controlled oral delivery of aspirin. Acta Chimica Slovenica, 61(4), 688–693. Retrieved from https://www.ncbi.nlm.nih.gov/pubmed/25551707. 25551707

[fsn32042-bib-0038] Toyonaga, T. , Matsuura, M. , Mori, K. , Honzawa, Y. , Minami, N. , Yamada, S. , Kobayashi, T. , Hibi, T. , & Nakase, H. (2016). Lipocalin 2 prevents intestinal inflammation by enhancing phagocytic bacterial clearance in macrophages. Scientific Reports, 6, 35014 10.1038/srep35014 27734904 PMC5062163

[fsn32042-bib-0039] Tsai, M.‐H. , Wu, C.‐H. , Lin, W.‐N. , Cheng, C.‐Y. , Chuang, C.‐C. , Chang, K.‐T. , Jiang, R.‐S. , Hsu, J.‐F. , & Lee, I.‐T. (2018). Infection with *Staphylococcus aureus* elicits COX‐2/PGE2/IL‐6/MMP‐9‐dependent aorta inflammation via the inhibition of intracellular ROS production. Biomedicine & Pharmacotherapy, 107, 889–900. 10.1016/j.biopha.2018.08.096 30257401

[fsn32042-bib-0040] Uysal, K. T. , Scheja, L. , Wiesbrock, S. M. , Bonner‐Weir, S. , & Hotamisligil, G. S. (2000). Improved glucose and lipid metabolism in genetically obese mice lacking aP2. Endocrinology, 141(9), 3388–3396. 10.1210/endo.141.9.7637 10965911

[fsn32042-bib-0041] Van Crombruggen, K. , Vogl, T. , Perez‐Novo, C. , Holtappels, G. , & Bachert, C. (2016). Differential release and deposition of S100A8/A9 proteins in inflamed upper airway tissue. European Respiratory Journal, 47(1), 264–274. 10.1183/13993003.00159-2015 26493794

[fsn32042-bib-0042] Vilaça, N. , Amorim, R. , Machado, A. F. , Parpot, P. , Pereira, M. F. R. , Sardo, M. , Rocha, J. , Fonseca, A. M. , Neves, I. C. , & Baltazar, F. (2013). Potentiation of 5‐fluorouracil encapsulated in zeolites as drug delivery systems for in vitro models of colorectal carcinoma. Colloids and Surfaces B: Biointerfaces, 112, 237–244. 10.1016/j.colsurfb.2013.07.042 23988779

[fsn32042-bib-0043] Wada, R. , Kawamura, C. , Inoue, S. , Watanabe, K. , Kaimori, M. , & Yagihashi, S. (2001). Granulomatous colitis associated with botryomycosis of *Propionibacterium acnes* . Archives of Pathology and Laboratory Medicine, 125(11), 1491–1493. 10.1043/0003-9985(2001)125<1491:GCAWBO>2.0.CO;2 11698011

[fsn32042-bib-0044] Wang, Y. , Liu, S. , Li, B. , Jiang, Y. , Zhou, X. , Chen, J. , & Cheng, L. (2019). *Staphylococcus aureus* induces COX‐2‐dependent proliferation and malignant transformation in oral keratinocytes. Journal of Oral Microbiology, 11(1), 1643205 10.1080/20002297.2019.1643205 31448061 PMC6691923

[fsn32042-bib-0045] Xavier, R. J. , & Podolsky, D. K. (2007). Unravelling the pathogenesis of inflammatory bowel disease. Nature, 448(7152), 427–434. 10.1038/nature06005 17653185

[fsn32042-bib-0046] Zarkovic, N. , Zarkovic, K. , Kralj, M. , Borovic, S. , Sabolovic, S. , Blazi, M. P. , & Pavelic, K. (2003). Anticancer and antioxidative effects of micronized zeolite clinoptilolite. Anticancer Research, 23(2B), 1589–1595. Retrieved from https://www.ncbi.nlm.nih.gov/pubmed/12820427. 12820427

[fsn32042-bib-0047] Zhang, J. , Dou, W. , Zhang, E. , Sun, A. , Ding, L. , Wei, X. , Chou, G. , Mani, S. , & Wang, Z. (2014). Paeoniflorin abrogates DSS‐induced colitis via a TLR4‐dependent pathway. American Journal of Physiology. Gastrointestinal and Liver Physiology, 306(1), G27–36. 10.1152/ajpgi.00465.2012 24232001 PMC3920084

