# Supplementary material for: Zeolite‐containing mixture alleviates microbial dysbiosis in dextran sodium sulfate‐induced colitis in mice
Source: Food Sci Nutr. 2020 Nov 30;9(2):772–80. doi: 10.1002/fsn3.2042 (PMC7866626; doi:10.1002/fsn3.2042)
Supplement: Supplementary file 1 — TableS1‐S2 [file FSN3-9-772-s001.docx]

**Table S1 (A).** Composition of the 0.8% zeolite-containing mixture (HY) added diet

| **Ingredients** | **Control Diet (AIN-93G)** | **Zeolite-Containing Mixture 0.8%** |
| --- | --- | --- |
| Cornstarch | 39.75% | 38.95% |
| Casein (85.7% protein) | 20% | 20% |
| Detrinized cornstarch | 13.2% | 13.2% |
| Sucrose | 10.25% | 10.25% |
| Soybean oil (no additives) | 7% | 7% |
| Fiber | 5% | 5% |
| Mineral mix (AIN 93G-MX) | 3.5% | 3.5% |
| Vitamin mix (AIN 93G-MX) | 1% | 1% |
| L-Cystine | 0.3% | 0.3% |
| Zeolite-containing mixture (HY) | 0 | 0.8% |

**Table S1 (B).** Composition of zeolite-containing mixture (HY, Hydryeast^®^)

| **Ingredients** | **Percentage** |
| --- | --- |
| Azumaceramics * | 47.6% |
| Citric acid | 33.3% |
| Red rice yeast (monascus) | 16.7% |
| Calcium stearate | 2.4% |

*: Azumaceramics is a mixture of zeolite and oyster shell burned under high temperature.

**Table S2.** Oligonucleotide sequences used in the real-time PCR analysis

| **Gene Symbol** | **Full name** |  | **Oligonucleotide sequence** |
| --- | --- | --- | --- |
| *Cox2* | cytochrome coxidase subunit 2 | F | AAGCCGAGCACCTTTGGAG |
|  |  | R | ATTGATGGTGGCTGTTTTGGTAG |
| *Sod2* | superoxide dismutase 2 | F | CTCCTGCTTGAGGAGTGCTT |
|  |  | R | CGGACAGGCCCTACCTACTT |
| *S100a8* | S100 calcium binding protein A8 | F | GGAAATCACCATGCCCTCTA |
|  |  | R | ATCACCATCGCAAGGAACTC |
| *S100a9* | S100 calcium binding protein A9 | F | CAGCATAACCACCATCATCG |
|  |  | R | GTCCTGGTTTGTGTCCAGGT |
| *Lcn2* | lipocalin 2 | F | CACGGACTACAACCAGTTCG |
|  |  | R | TCCTTGAGGCCCAGAGACT |
